# Attention Deficit Disorder with Hyperactivity Symptoms in Early-Treated Phenylketonuria Patients

**Published:** 2020

**Authors:** Mayara Thays BECKHAUSER, Marcella BEGHINI MENDES VIEIRA, Betine MOEHLECKE ISER, Gisele ROZONE DE LUCA, Marcelo RODRIGUES MASRUHA, Jaime LIN, Emilio LUIZ STRECK

**Affiliations:** 1 Department of Neurology- Hospital Santa Isabel - Blumenau – Santa Catarina- Brazil; 2 Department of Biology, Health and Social Sciences, Universidade do Sul de Santa Catarina (UNISUL) -Tubarão - Santa Catarina - Brazil; 3 Department of Pediatric Neurology - Universidade Federal de São Paulo (UNIFESP) - São Paulo - São Paulo - Brazil; 4 Programa de Pós Graduação em Ciências da Saúde (PPGCS) - Laboratório de Pesquisa de Doenças Neurometabólicas - Universidade do Extremo Sul Catarinense (UNESC) -Criciúma - Santa Catarina - Brazil

**Keywords:** Phenylketonuria, Phenylalanine, Attention Deficit, Disorder with Hyperactivity

## Abstract

**Section Title:**

Objectives To assess the presence of symptoms consistent with Attention Deficit Disorder with Hyperactivity (ADHD) in all patients with early-treated phenylketonuria (PKU) in the State of Santa Catarina in southern Brazil.

**Materials & Methods:**

All of the patients diagnosed with PKU by newborn-screening tests, with ages varying from 6 to 18 years and who started treatment before 60 days of life and presented phenylalanine levels consistently below 6 mg/dL throughout treatment, were included. The subjects were invited to complete a questionnaire that collected sociodemographic, gestational and clinical data. ADHD symptoms were assessed using the revision of the Swanson, Nolan and Pelham Questionnaire.

**Results:**

A total of 34 patients were evaluated, who were 53% male and 94% white and had an average age of 12 years, and 15% were born premature. According to the Swanson, Nolan and Pelham Questionnaire, 13 patients (38%) met the diagnostic criteria for ADHD, with 2 patients having the inattentive type, 6 patients having the hyperactive or impulsive type and 1 patient having the oppositional defiant disorder type.

**Conclusion:**

Although the patients with PKU were regularly treated from birth, there was a high prevalence of symptoms consistent with ADHD. A pathophysiological interface that involves the dopamine metabolic pathway may exist between the two conditions.

## Introduction

Phenylketonuria (PKU) is a genetic disorder that involves the ineffective conversion of Phe (phenylalanine) into tyrosine and consequently causes a deficit in the production of precursors, one of which is dopamine (DA) [1-6]. Neurobiologically, Attention Deficit Disorder with Hyperactivity (ADHD) appears to be closely related to the dopaminergic neurotransmission system. DA is a neurotransmitter that has a key role in attention and concentration. Moreover, cortical dopaminergic pathways act in mediating cognitive functions, such as learning, maintaining attention and concentration [3,4]. Individuals with PKU and ADHD seem to have low levels of DA in common, causing the hypodopaminergic state found in both disorders to be a possible link between these two conditions, and individuals with PKU could have a greater predisposition to the development of ADHD.

The prefrontal cortex is especially sensitive to low levels of DA, and it is speculated that even moderate elevations of Phe may result in low levels of DA in the central nervous system. ADHD and PKU are therefore theoretically linked by low levels of available DA in the prefrontal cortex [7-9]. Many authors have questioned the existence of a pathophysiological relationship between PKU and ADHD and emphasize the importance of additional studies that may further elucidate the relationship between these entities [10]. Despite the existence of theoretical background, there are few reports studying the linkage between ADHD and PKU. The aim of this study was to verify the presence of ADHD symptoms in patients with early-treated PKU. 

## Materials & Methods

A cross-sectional study assessing ADHD symptoms in every early-treated PKU patient living in the State of Santa Catarina was conducted in 2012. The study was performed at the Hospital Infantil Joana de Gusmão, the largest pediatric hospital in the State of Santa Catarina. Located at Florianópolis, the capital city of Santa Catarina, the Hospital Infantil Joana de Gusmão is a tertiary center for metabolic disorders. Every case of PKU detected by newborn-screening tests is referred to this hospital.

*Patients:*

Every PKU patient diagnosed by a neonatal-screening test in the State of Santa Catarina was enrolled in this study. Of those patients, every individual between 6 and 18 years old who was regularly treated since birth, according to the “Brazilian Phenylketonuria Clinical and Therapeutic Guidelines”, was included in this study [11]. 

Every PKU patient who started treatment after 60 days of age, who failed to maintain Phe levels below 6 mg/dL or who failed to adhere to regular medical follow-ups were excluded.

*Procedures:*

During regular medical appointments, the patients’ legal representatives, who were usually the patients’ parents, were asked to complete questionnaires regarding epidemiological data (age, gender, income and educational degree), clinical data (familial consanguinity, pregnancy and birth) and ADHD symptoms. The Portuguese version of the “Swanson, Nolan and Pelham Questionnaire (SNAP-IV)” was used [12].

*ADHD:*

The diagnosis of ADHD is essentially clinical, based on clear and well-defined operational criteria derived from classification systems such as the Diagnostic and Statistical Manual of Mental Disorders , Fourth Edition (DSM-IV) [13].

According to the DSM-IV, for the diagnosis of ADHD, it is essential to identify the presence of symptoms consistent with ADHD that present before the age of 7 in at least two different contexts (e.g., at home and at school) and are associated with evident damage in school, social or family life and to determine whether those existing symptoms are not better explained by the presence of another psychiatric disorder. A confirmation of the diagnosis can only be achieved through proper neurological or psychiatric investigation [14].

In this study, the Portuguese version SNAP-IV was used for the standardization of ADHD symptoms [12].

The SNAP-IV is a questionnaire containing 26 items aimed at parents and teachers and related to ADHD symptoms. The items are subdivided into scales related to the symptoms of inattention (items 1 to 9), hyperactivity or impulsivity (items 10 to 18) and oppositional defiant disorder (ODD; 19 to 26). Each question about a symptom can be answered in four different ways: “not at all”, “just a little”, “quite a bit” or “very much”. Only the presence of six or more responses of “quite a bit” or “very much” on the items related to inattention and/or hyperactivity indicate the presence of ADHD symptoms [12].

When there are at least six items marked as “quite a bit” or “very much” among items 1 to 9 of the SNAP-IV, there are more symptoms of inattention than expected in a child or adolescent, and the individual has symptoms suggestive of the inattentive subtype of ADHD [12,15].

If there are at least six items marked as “quite a bit” or “very much” among items 10 to 18 of the SNAP-IV, then there are more symptoms of hyperactivity or impulsivity than expected in a child or adolescent, thus identifying the hyperactive or impulsive subtype of ADHD [12,15,16].

We included items related to the DSM-IV criteria for ODD, corresponding to items 19 to 26 of the SNAP-IV, because ODD increases the risk of the emergence of antisocial behavior in children and adolescents and is often present in those individuals with ADHD [12].

*Statistical Analysis:*

Statistical analysis was performed using EpiInfo version 3.5.3 for Windows.

Results are presented as absolute and relative frequencies for qualitative variables and as the mean value and standard deviation for quantitative variables. Differences in proportions were assessed by a chi-square or Fisher’s exact test, with a significance level of 5%. A possible association between risk factors and ADHD was verified by the prevalence ratio (PR), with a confidence interval (CI) of 95%. 

## Results

In 2012, the State of Santa Catarina had a total of 142 patients with a confirmed diagnosis of PKU, of which 77 were diagnosed and treated from birth. Among those individuals, a total of 34 patients were considered to be eligible for our study.

*Sociodemographic and Epidemiological Aspects:*

**Figure 1:**
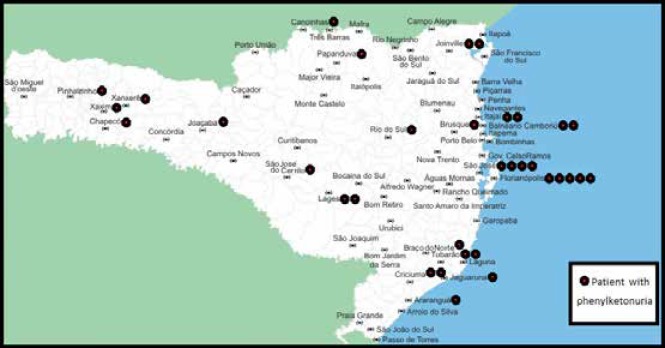
Distribution of patients with phenylketonuria treated early in the State of Santa Catarina according to the city of birth (n=34)

Adapted from “Guia Turístico e Cultural do Estado de Santa Catarina”. Web site: http://www.sctur.com.br/images/mapa_sc_2008g.jpg. Acessed in March 14, 2013.

Regarding the city of birth, there is a homogeneous distribution across the State of Santa Catarina, with the 34 PKU patients spread across all regions of the state, as observed in detail in Figure 1.

Regarding the epidemiological variables, as shown in Table 1, there was a slight predominance of men (53%) and white patients (94%) in the sample. The mean age was 12 years. Considering children who were 12 years old or less and adolescents who were older than 12 years old, our sample consisted of 19 children (56%) and 15 adolescents (44%). Regarding family income, we observed that most parents of PKU patients earned up to five times the minimum wage.

It was observed that only 2 patients had a history of parental consanguinity. In one case, the parents were cousins​​, and in another, the parents were an uncle and niece. Most patients were born by cesarean delivery and at term. Most patients received regular treatment, consisting of a specific diet for PKU (foods with Phe restriction) and using special protein formulas (with Phe restriction). A minority of patients only received dietary treatment but still managed to successfully keep their blood Phe levels below 6 mg/dL (Table 1).

*Education Level:*

Regarding the education of the PKU patients, 26 patients (76%) were up-to-date with their age-appropriate class, without grade repetition. 

Concerning the higher parents’ education, half of the parents had a complete or incomplete university education (Table 2).

*ADHD:*

Taking into account the criteria for clinical symptoms suggestive of ADHD, i.e., considering six or more answers of “quite a bit” or “very much” on the SNAP-IV questionnaire as positive for ADHD, 13 patients (38%) in our sample showed symptoms suggestive of ADHD.

Among the patients with symptoms suggestive of ADHD, 2 patients (15%) could be classified as having the inattentive subtype of ADHD, whereas 6 patients (46%) could be classified as having the hyperactive or impulsive ADHD subtype according to the SNAP-IV. Only 1 patient fit the ODD criteria and also showed symptoms suggestive of ADHD.

A statistical comparison of the data can be viewed in detail in Table 3. When comparing the prevalence of symptoms suggestive of ADHD in relation to gender, we found that 6 patients (46%) were female, whereas 7 patients (54%) were male. There was no statistical significant relationship between sex and the prevalence of positive symptoms of ADHD. 

Considering prematurity and positive ADHD symptoms, 2 patients (15%) were premature, whereas 11 patients (85%) were born at term. Thus, in this case, prematurity was not related to the increased presence of symptoms suggestive of ADHD (Table 3).

Regarding parental consanguinity and positive symptoms of ADHD, we found that all patients with consanguineous parents (n=2) had symptoms consistent with ADHD. Of the 32 patients whose parents were not consanguineous, 11 subjects (34%) had symptoms consistent with ADHD. When the PR between symptoms consistent with ADHD and the presence or absence of consanguineous parents was calculated, it was observed that the prevalence of ADHD symptoms was approximately three times higher in patients with consanguineous parents than in patients without consanguinity (PR = 2.9, 95% CI 1.8 to 4.7, Table 3).

Regarding the type of delivery, 5 patients (38%) with symptoms suggestive of ADHD were born vaginally, and 8 (62%) were born through a cesarean. The ADHD diagnosis prevalence rate of those individuals who underwent a normal birth compared with those patients who were born through a cesarean delivery was 1.14 (95% CI 0.48 to 2.73), and there was no statistical significant association between the type of delivery and ADHD diagnosis (Table 3).

## Discussion

Individuals with PKU and ADHD seem to have low levels of DA in common, causing the hypodopaminergic state found in both disorders to be a possible link between these two conditions, and individuals with PKU could have a greater predisposition to the development of ADHD.

For the first time in Brazil, this study assessed the relationship between PKU, a rare genetic and neurometabolic disease, with ADHD, the most common neurobehavioral condition affecting the pediatric population. We believe that these two conditions can be comorbid.

**Table 1:** Epidemiological variables for patients with phenylketonuria treated early in the State of Santa Catarina (n=34)

Variable		Frequency (n)	Percentage (%)
Gender	Female Male	16 18	47 53
Age (years)	6 to 12 13 to 18	19 15	44 56
Skin color Race	White Brown	32 2	94 56
Parental consanguinity	Yes No	2 32	6 94
Child-birth	Normal/vaginal Cesarean	12 22	35 65
Prematurity^a^	Yes No	5 29	15 85
Family income^b^	Until 5 6 to 10 More than 10	23 10 1	67 30 3
Treatment	Diet and protein formula Diet	26 8	76 24

^a^A premature baby was considered to be a baby born before 37 complete weeks of gestation.

^b^Income is expressed in minimum wages, for which one wage corresponds to 622 BRL ($315.73 as of 03/15/2013).

**Table 2:** Education level of patients with phenylketonuria and parents’ education (n=34).

Variable		Frequency	Percentage
Patients’ education	Without repetition Lost 1 year Lost 2 years Lost more than 3 years	26 6 1 1	76% 18% 3% 3%
Parents' education	Elementary school High school Incomplete university Complete university	10 7 11 6	29% 21% 32% 18%

**Table 3:** Relationship between characteristics in patients with phenylketonuria and symptoms suggestive of Attention Deficit Disorder with Hyperactivity (n=13)

	N	%	*p value*
Gender			
Female	6	46	0.93^a^
Male	7	54	
Gestational age			
Premature	2	15	0.64^b^
Term	11	85	
Parental consanguinity			
Yes	2	15	0.13^b^
No	11	85	
Childbirth			
Normal/vaginal	5	38	0.52^b^
Cesarean	8	62	

^a^Chi-square test, significance level of 5%.

^b^Fisher’s exact test, significance level of 5%.

The term “comorbidity” was initially described by Feinstein to describe the association of two morbid conditions in the same person [17]. More recently, this term has been used with a more restricted meaning, referring to the non-random association of two or more morbid conditions in a single individual [18].

The proper study of comorbid conditions is of extreme importance to several aspects, yielding implications for the correct diagnosis of both conditions, allowing the creation of new therapeutic opportunities and possibly providing clues to understanding the pathophysiology of diseases.

The present study also allowed the study of a highly specific population: patients with PKU diagnosed from birth who started treatment early and, most importantly, are continuing to receive proper treatment.

In our study, we found the presence of symptoms consistent with ADHD in 13 patients (38.2%), most of whom were classified as having the hyperactive or impulsive subtype of ADHD. This prevalence is much higher than the prevalence verified in previous studies: 4 to 12% with ADHD in the general pediatric population of 6 to 12 years old [8,19]. In Brazil, Vasconcelos MM et al., who evaluated children between 6 and 15 years old, found a 17.1% prevalence of ADHD, mostly of the inattentive subtype [20].

A significant percentage of our sample lost at least 1 year in schooling. Antshel KM et al. claim that academic difficulties are relatively common in children and adolescents with PKU and may be a function of ADHD, executive function deficits or deficits in processing speed [4]. Moreover, the high prevalence of symptoms consistent with ADHD found in our sample of PKU patients suggests a link to the high repetition rate that we found. In a meta-analysis, Frazier TW et al. reported the average difference in academic performance between ADHD and control groups, by showing that young people with ADHD have a higher risk of failure and abandoning school [21]. Authors stressed that so far, no data have been reported on the association between ADHD and school outcomes in the PKU population [22].

Certain studies observed an association between the PKU and TDAH. A study by Antshel KM et al. examined a sample of 46 children with classical PKU (mean age: 10.8 years), several of these individuals began treatment late and thus were exposed to high levels of Phe. Using strict DSM-IV criteria, 6 children (13%) met the full criteria for ADHD, with a prevalence that was 2.5 times higher than in the general population in this study. All children were diagnosed with ADHD of the inattentive type. The results indicated that high levels of Phe are toxic to the neurological system that manages the executive and cognitive functions and that the duration of exposure to high levels of Phe can affect the expression of ADHD symptoms [23,24].

As in the current study, other authors have demonstrated an indirect association between PKU and attention disorders, as demonstrated by Arnold GL et al., who evaluated the prevalence of stimulant medication use by reviewing the medical records of a sample of 38 young people with classical PKU treated early and continuously (mean age: 11 years) [22]. In total, 19 of the parents of the young people with PKU (50%) reported that their child had significant symptoms of inattention. For 10 children, a stimulant medication for attentional dysfunction was prescribed, which, according to parental report, was effective. Furthermore, a significant relationship was found between high levels of Phe with symptoms of inattention and the increased use of stimulants [22].

Nothing has been found in the literature that is similar to the results of our study, in which only patients treated properly and early for PKU were selected and in which PKU’s association with ADHD was observed. Many authors emphasize the need for studies to more clearly and explicitly demonstrate the relationship between PKU and ADHD [16,23]. This study demonstrated a possible relationship between these two entities, reinforcing the theory that the hypodopaminergic state found in patients with PKU may contribute to the development of ADHD.

ADHD is a multifactorial condition with risk factors with genetic and environmental aspects [20]. The influence of environmental factors is highly accepted in the literature, and especially the relationship with premature birth and low parental education [25]. In our study, patients with symptoms consistent with ADHD were mostly born at term, and the patients’ parents had higher education in 50% of cases. Thus, we speculate that ADHD symptoms may be nearly entirely related to PKU.

A literature review conducted by Brumm VL et al. on psychological and psychiatric disorders and PKU reveals that the prevalence and severity of these problems are correlated with the time and degree of exposure to elevated Phe levels in the blood [26]. Children with poor metabolic control and those individuals with a late initiation of treatment are more likely to be affected and have more severe symptoms. The authors report that individuals with PKU, even if treated continuously, have an increased risk of presenting symptoms of inattention and distraction [26]. The current literature mostly emphasizes that most neurobehavioral symptoms found in patients with PKU who are diagnosed and treated early may be related to poor adherence to treatment or fluctuations in Phe levels throughout life [3].

In our study, however, we evaluated only patients diagnosed and treated early who undergo regular medical monitoring and always maintain their Phe levels as recommended. Thus, we suggest that the neurobehavioral symptoms cannot be fully explained by a failure in therapy. R Sharman et al. support this concept, suggesting that monitoring the levels of tyrosine, an important metabolite of the DA synthesis pathway, and its eventual replacement must be added to the therapeutic armamentarium of PKU [27].

In our study, certain limitations can be observed. It should be noted that other criteria are also needed for the diagnosis of ADHD. The SNAP-IV criteria must be accompanied by a neuropsychological evaluation of these patients, and other tests may be used to assist in diagnosis. To develop a more precise diagnosis, an investigation of the story of a child’s life is always appropriate for contextualizing symptoms.

In addition, because PKU is a rare condition, although we evaluated all patients in the State of Santa Catarina, we analyzed a relatively small population, preventing a more appropriate analysis of the data. Regarding the type of study, the impossibility of inferring causality from the studied factors from a cross sectional study is emphasized.

**In Conclusion, **Our study reinforced the theory that the hypodopaminergic state found in patients with PKU, although appropriately treated, may contribute to the development of other conduct disorders, and particularly ADHD. Although patients are regularly treated from birth, PKU can be an important risk factor for the development of ADHD. Studies such as this one suggest an interface between the two pathophysiological conditions that involves dopamine metabolism. It is of great importance that professionals who manage PKU patients are aware of all of the neuropsychological symptoms that an individual might present to ensure more effective therapy.
